# Excessive risk of second primary cancers in young‐onset colorectal cancer survivors

**DOI:** 10.1002/cam4.1437

**Published:** 2018-03-13

**Authors:** Xingkang He, Wenrui Wu, Yu'e Ding, Yue Li, Jianmin Si, Leimin Sun

**Affiliations:** ^1^ Department of Gastroenterology Sir Run Run Shaw Hospital Zhejiang University Medical School Hangzhou China; ^2^ Institute of Gastroenterology Zhejiang University (IGZJU) Hangzhou China; ^3^ Department of Microbiology Tumor and Cell Biology Karolinska Institute 171 77 Stockholm Sweden; ^4^ State Key Laboratory for Diagnosis and Treatment of Infectious Diseases The First Affiliated Hospital School of Medicine Zhejiang University Hangzhou China

**Keywords:** Colorectal cancer, prevention, second primary cancer, SEER, young survivors

## Abstract

With an increasing trend of patients with young‐onset colorectal cancer (CRC), risks of second primary cancers (SPCs) among them become a concerning issue. We aimed to define the detailed risk and site‐distributed patterns of SPCs in young CRC individuals (age ≤50). A population‐based cohort were identified from the Surveillance, Epidemiology, and End Results database between 1973 and 2013. Standardized incidence ratios (SIRs) and absolute excess risk (AER) were calculated to assess the risk for SPCs compared with the general population. A total of 44,106 patients, including 3245 (7.4%) the young and 40,861 (92.6%) the old, developed 50,679 secondary malignancies subsequently. With increased age, the risk of secondary cancers gradually decreased. A significant 44% excess risk of SPCs was observed in the young (SIR = 1.44, AER = 34.23), while a slightly increased risk was noted in the old (SIR = 1.02, AER = 4.29). For young survivors, the small intestine (SIR = 8.49), bile ducts (SIR = 3.77), corpus, and uterus (SIR = 2.45) were the most common sites of SPCs. Significantly, excess SIRs in the young were persisted regardless of other factors. For the young, secondary cancer‐related deaths were responsible for 51.2% of overall deaths and secondary stomach, liver and bile, pancreas cancers were top three causes. An excessive risk of SPCs existed in young CRC survivors, and this trend was consistent among different subgroups. We hope our findings may inform future targeted screening strategies among young‐onset CRC survivors.

## Introduction

Colorectal cancer (CRC) remains the third most common malignancies among men and women in the United States according to Colorectal Cancer Statistics, 2017 1. During past several decades, overall incidence and mortality of CRC are declining significantly across population over the age of 50 years in the United States. It may largely attribute to the introduction and implement of cancer screening, healthier lifestyle, and advanced therapies 2, 3, 4.

According to Survivorship Statistics released by American Cancer Society, it is estimated that more than 15 million survivors are living in the United States with a previous colorectal cancer diagnosis in 2016 5. A large body of literatures reported the increased risk of second primary malignancies among population with certain prior cancers, including CRC 6, 7, 8, 9, 10. Several population‐based studies had validated that a history of prior CRC was a known risk factor for developing second malignancies subsequently in relation to the general population 11, 12, 13, 14. Although the clear mechanism underlying this trend remained unknown, a possible combination of genetic predisposition, risk exposures, and cytotoxic effects of chemotherapy, radiotherapy could be at play 15, 16, 17, 18, 19, 20, 21. Currently, the worrying trend of increasing rate of young‐onset CRC has been reported and warned by recent literature 22, 23, 24. Considering longer life expectancy of young patients, it is plausible that the likelihood of developing a second primary malignancy later in life would become larger. Several studies had indicated that young CRC survivors are predisposed to subsequent cancers 25, 26, 27.

Therefore, the issue regarding the risk of SPCs in the young needs to be further explored. We performed a retrospective population‐based study to quantify the risk and characterize site‐distribution patterns of SPCs in the young to address this important gap. From a clinical perspective, it is critical to understand and characterize patterns of SPCs among young survivors in order to provide the appropriate surveillance strategies for these populations.

## Method

### Data source and population

We conducted this retrospective study by utilizing the National Cancer Institute's (NCI's) Surveillance, Epidemiology, and End Results (SEER) database, which approximately cover 28% of the United States population 28. The SEER program routinely collects the high‐quality demographic, clinical, and follow‐up information from 18 cancer registries. Current data retrieved from SEER 9 Regs Research Data, Nov 2015 Sub (1973–2013) <Katrina/Rita Population Adjustment> (nine registries including San Francisco–Oakland SMSA, Connecticut, Detroit, Hawaii, Iowa, New Mexico, Seattle, Utah, Atlanta) 29. Patients age ≥18 years, who were diagnosed with first primary microscopically confirmed colon and rectum cancer (SEER code, site recode B ICD‐O‐3/WHO 2008) between 1973 and 2013, were included in this selected cohort. We excluded cases with CRC that was identified by death certificate or autopsy only. To insure that synchronous primary malignancies were not classified as second primary cancers, we included cases with at least 6‐months latency restriction (period between the time of primary CRC diagnosis and the time of a second cancer diagnosis). We also conducted sensitivity analyses further excluding cases diagnosed within CRC before a year. Considering the fact that average‐risk screening is recommended to begin at 50 years of age by American Cancer Society, we defined patients aged ≤50 years as young survivors and cases aged >50 years as old survivors at the time of diagnosis. For tumor subsites, we divided patients into three groups by primary site‐labeled: proximal colon (C18.0, C18.2–C18.4), distal colon (C18.5–C18.7), rectum (C19.9, C20.9). Other sites (C18.8, C18.9, C26.0) were coded as unclear. Our study was approved by the review board of Zhejiang Institute of Gastroenterology, Sir Run Run Shaw Hospital, China.

### Statistical analyses

Multiple primary‐standardized incidence ratios (MP‐SIR) sessions of SEER*Stat version 8.3. (SEER Program, National Cancer Institute) were adopted to calculate standardized incidence ratios (SIRs) and absolute excess risk (AER). The SIR, also known as the relative risk, is calculated by dividing the observed number of second cancers by the expected number of cancers [observed/expected (O/E) ratio] in the general population without primary malignancies. AER was defined as the excess cancer per 10,000 persons per year, which is an absolute measure of the clinical burden of additional cancer 30. More detailed information about SEER*Stat software and the methods are available on the SEER‐registry website (https://seer.cancer.gov/resources/). SIRs for subgroup analysis were further stratified by gender (male and female), race (White, Black, American Indian/Alaska Native, Asian or Pacific Islander), calendar year (1973–1983, 1984–1993, 1994–2003, 2004 + ), latency period after first CRC diagnosis (6–11, 12–59, 60–119, 120 + months), SEER stage (localized, regional, distant), cancer subsite (proximal, distal colon, rectum), radiotherapy (radiation, no radiation). Additionally, we also assessed the cause of death among young survivors to elucidate influence of second cancers in their prognosis. We defined the cause of death based on the cause of death (COD) to site recode in the SEER. Furthermore, we adopted standardized mortality ratios (SMRs) to compar the relative risk of death for patients with CRC in relation to general US population. All statistical tests of significance were two‐sided, and a *P* value <0.05 was considered statistically significant.

## Results

Totally, 44,106 CRC survivors, including 3245 (7.4%) young and 40,861 (92.6%) old survivors, were included in this study. They developed 50,679 SPCs at least 6 months after the original diagnosis. Characteristics of those patients were summarized in Table 1. The total observation period was 2,474,945.72 person‐years with a mean of 7.31 person‐years at risk.

**Table 1:** Baseline characteristics of patients with subsequent primary cancers with prior diagnosis of colorectal cancer

Numbers of patients (%) *N* = 44,106		
Characteristic	Young patients aged ≤50 years *N* = 3245	Old patients aged >50 years *N* = 40,861
Gender		
Male	1735 (53.5%)	23,779 (58.2%)
Female	1510 (46.5%)	17,082 (41.8%)
Calendar year		
1973–1983	1100 (33.9%)	10,897 (26.7%)
1984–1993	918 (28.3%)	13,688 (33.5%)
1994–2003	780 (24.0%)	11,416 (27.9%)
2004–2013	447 (13.8%)	4880 (11.9%)
Race		
White	2504 (77.2%)	35,184 (86.1%)
Black	419 (12.9%)	3136 (7.6%)
AI/AN	29 (0.9%)	119 (0.3%)
AP	291 (9.0%)	2406 (5.9%)
Unknown	2 (0.1%)	16 (0.1%)
SEER stage		
Localized	1584 (48.8%)	22,580 (55.1%)
Regional	1382 (42.6%)	15,765 (38.6%)
Distant	181 (5.6%)	1316 (3.2%)
Unstaged	98 (3.0%)	1200 (2.9%)
Grade		
Well differentiated	422 (13.0%)	5707 (14.0%)
Moderately differentiated	1520 (46.8%)	21,722 (53.2%)
Poorly differentiated	433 (13.3%)	4724 (11.6%)
Undifferentiated	27 (0.8%)	270 (0.7%)
Unknown	843 (26.0%)	8438 (20.7%)
Primary cancer subsites		
Proximal	1124 (34.6%)	14,887 (36.4%)
Distal colon	1038 (32.0%)	14,079 (34.4%)
Rectum	1012 (31.2%)	11,305 (27.7%)
Unclear	71 (2.2%)	590 (1.4%)
Therapy		
Radiation	2795 (86.1%)	37,073 (90.7%)
No radiation	426 (13.1%)	3535 (8.7%)
Unknown	24 (0.7%)	253 (0.6%)

AI/AN, American Indian/Alaska Nativel; AP, Asian or Pacific Islander.

**Figure 1:**
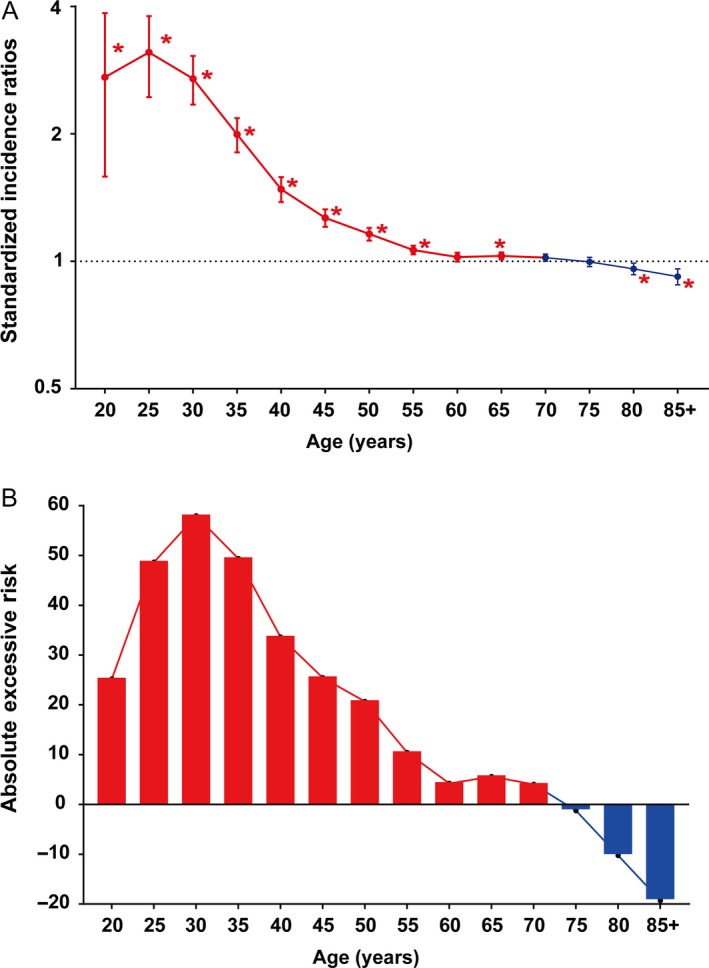
Standardized incidence ratio and absolute excess risk for second primary cancers of all sites by age among colorectal survivors. (A) standardized incidence interval; (B) absolute excess risk. *P <* *0.05 (compared with general population).

**Figure 2:**
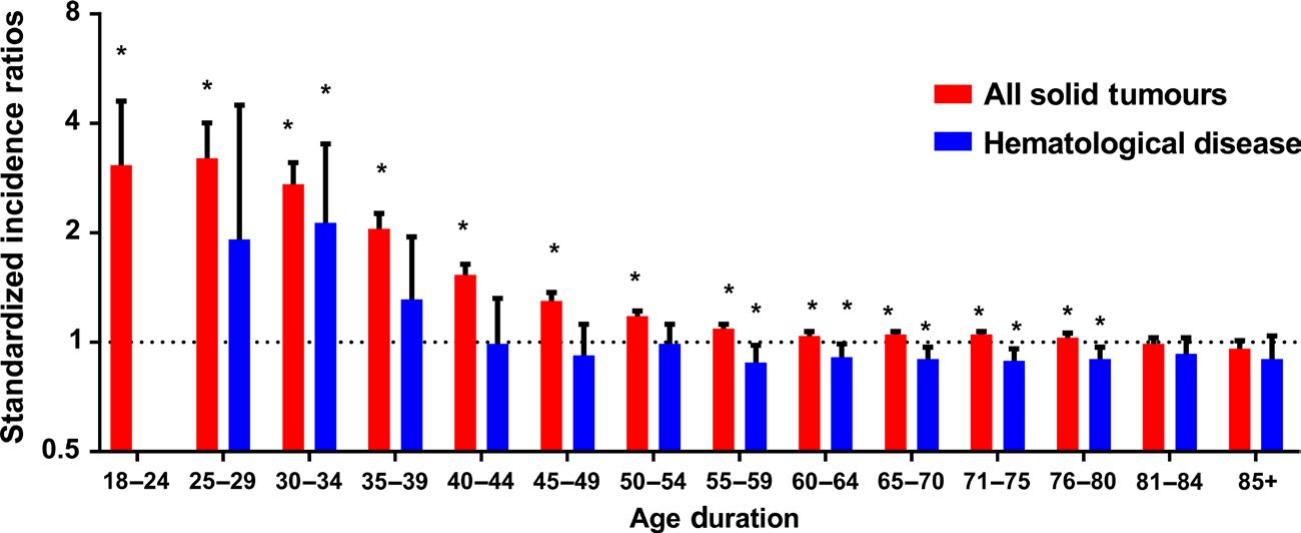
Standardized incidence ratios for all second solid tumor and hematological diseases by age duration. *P <* *0.05 (compared with general population).

**Figure 3:**
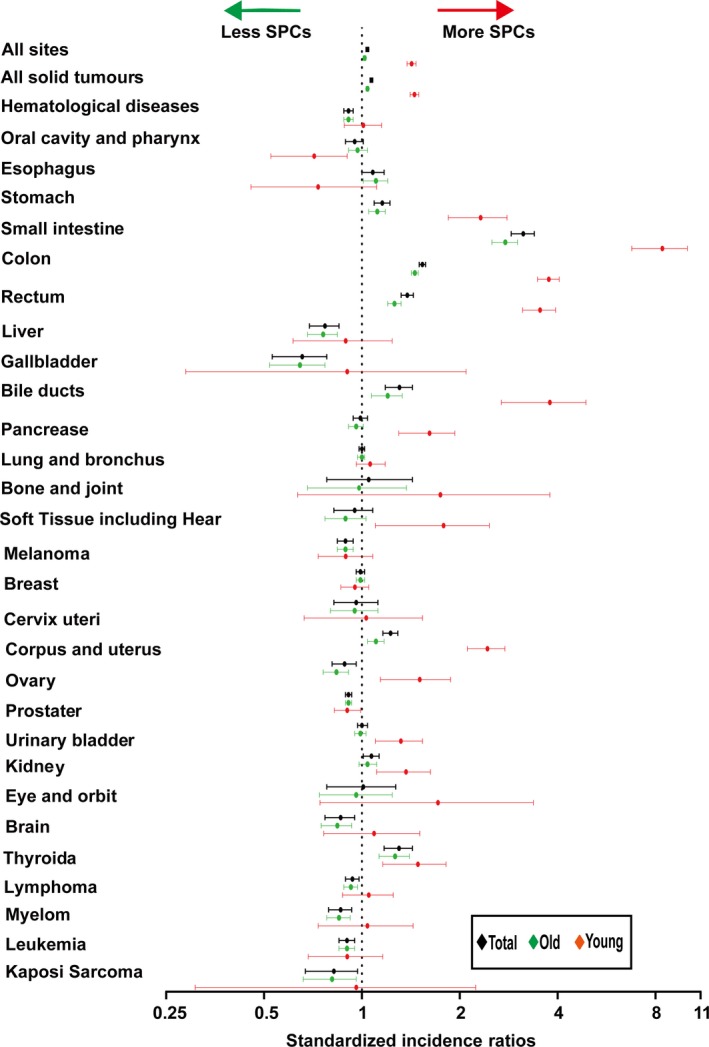
Standardized incidence ratios for subsequent primary malignancies in different sites by age group. SPCs, second primary cancers. *P <* *0.05 (compared with general population).

Regardless of detailed sites of secondary cancers, the overall SIR was inversely associated with age of onset (Fig. 1A). The absolute excess risk was also markedly elevated for young population, especially for individuals aged between 25 and 45 years (Fig. 1B). Compared with general population, young survivors were at increased risk of all solid tumors, while no significant SIR of hematological disease was observed (Fig. 2). The SIRs of all sites, all solid tumors, hematological disease were 1.44, 1.47, 1.01 times higher for young survivors and 1.02, 1.05, 0.91 times for old groups (Table S1). The AER of all sites, all solid tumors, hematological disease were 34.23, 33.07, 0.06 excess risk per 10,000 persons per year for young groups, while 4.29, 8.92, −1.75 excess risk for old patients (Table S1). For site‐distribution patterns of SPCs, there were several significant differences across young and old survivors (Fig. 3). In the young, the excess risk was observed for subsequent noncolonic cancers of small intestine (SIR, 8.49, 95% CI = 6.93–10.28), bile ducts (SIR, 3.77, 95% CI = 2.76–5.03), corpus and uterus (SIR, 2.45, 95% CI = 2.14–2.79, for female), stomach (SIR, 2.31, 95% CI = 1.86–2.83), soft tissue including heart (SIR, 1.59, 95% CI = 1.02–2.37), pancreas (SIR, 1.62, 95% CI = 1.32–1.97), ovary (SIR, 1.51, 95% CI = 1.17–1.93, for female), thyroid (SIR, 1.48, 95% CI = 1.18–1.84), kidney (SIR, 1.37, 95% CI = 1.13–1.65), urinary bladder (SIR, 1.32, 95% CI = 1.12–1.56) (Table S1). In contrast, a slightly increased risk for SPCs of small intestine (SIR, 2.76, 95% CI = 2.52–3.02), thyroid (SIR, 1.26, 95% CI = 1.13–1.40), bile ducts (SIR, 1.20, 95% CI = 1.07–1.33), stomach (SIR, 1.11, 95% CI = 1.05–1.18), corpus and uterus (SIR, 1.10, 95% CI = 1.04–1.17), esophagus (SIR, 1.10, 95% CI = 1.01–1.20) were found in old population (Table S1).

**Figure 4:**
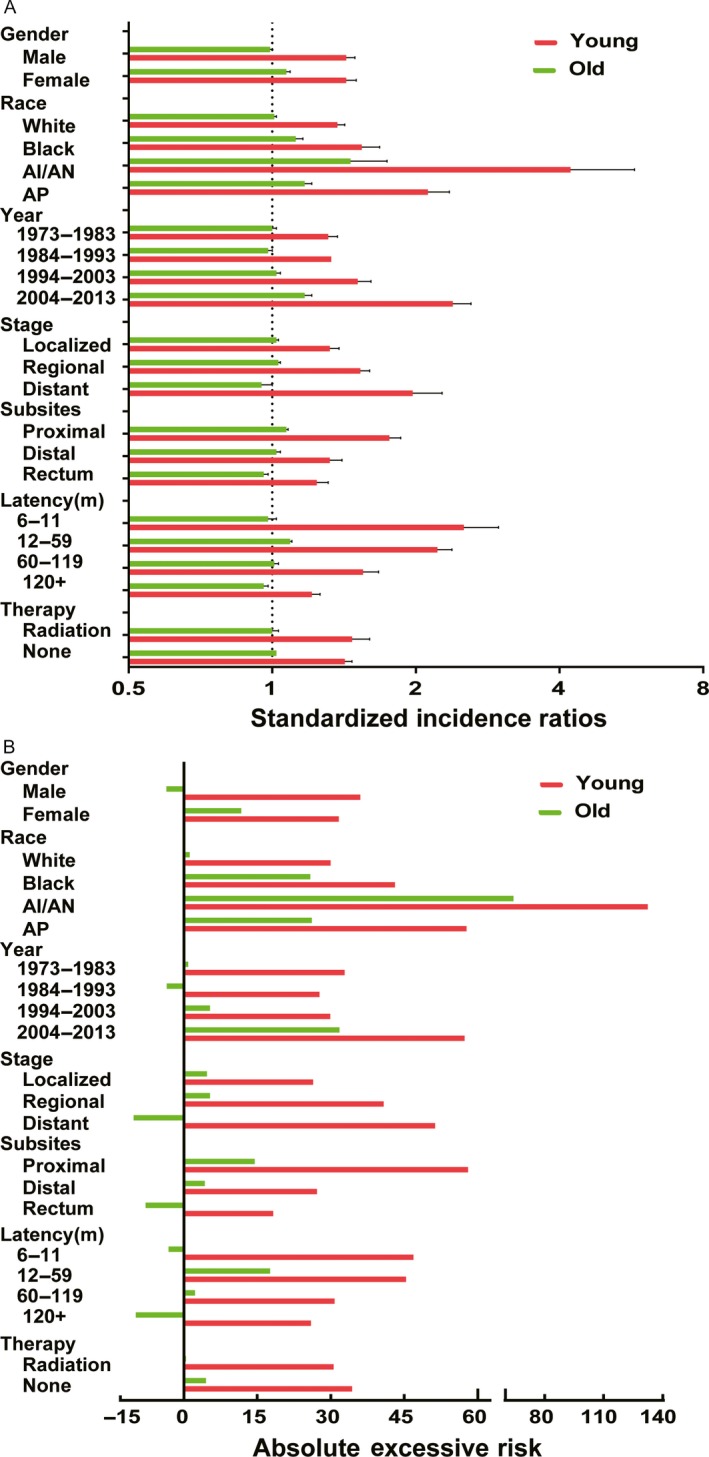
Standardized incidence ratios and absolute excess risk for subsequent cancers in different sites among young and old survivors by gender, race, year, stage, subsites, latency, therapy group. (A) standardized incidence interval; (B) absolute excess risk. m, month, AI/AN, American Indian and Alaska Native; AP, Asian or Pacific Islander. *P < 0.05 (compared with general population).

Furthermore, we conducted subgroup analysis to determine whether the SIRs of SPCs were consistent in subgroups. The significant increased SIRs, AER of SPCs were observed in young population regardless of different variables (Fig. 4A and B). For different gender, young male survivors were at significant risk for SPCs of small intestine compared with female young survivors (Table S2). For different race, the highest risk for SPCs belongs to American Indian/Alaska Native (AI/AN) in both young and old survivors (Table S3). In young survivors, compared with White and Black people, AP and AI/AN had dramatically higher risk of SPCs and the magnitude was huge. The most common sites for SPCs in young AI/AN patients were colon, stomach, and ovary, which was in contrast with old AI/AN patients. For different calendar periods, the risk of SPCs in young survivors elevated significantly and gradually from 1973–1983 to 2004–2013 (Table S4). Among old population, the elevated risk was only noted during the period between 1994–2003 and 2004–2013. For different stages, the risk of all sites SPCs increased gradually among young survivors from localized, regional to distant stage, whereas the risk for SPCs of all sites significantly reduced in old survivors (Table S5). This might be due to short survival period of old patients with CRC. For different subsites, in young survivors, the highest SIR of all sites SPCs was observed in proximal colon cancer compared with distal colon and rectum cancer (Table S6). In old survivors, patients with rectum cancer even had decreased overall risk of SPCs. For different latency, significant risk of SPCs was centered in initial 5 years after primary diagnosis among young survivors and even after 10 years (Table S7), the risk still be significantly higher for SPCs of small intestine, colon, rectum, kidney, stomach, bile ducts, urinary bladder. In old population, slightly increased risk of SPCs was only centered in initial 5 years and decreased with time. For patients who did and did not receive radiation, there was no different risk of all sites SPCs in young groups. Receipt of radiation did not lead to a higher risk of SPCs (Table S8).

**Figure 5:**
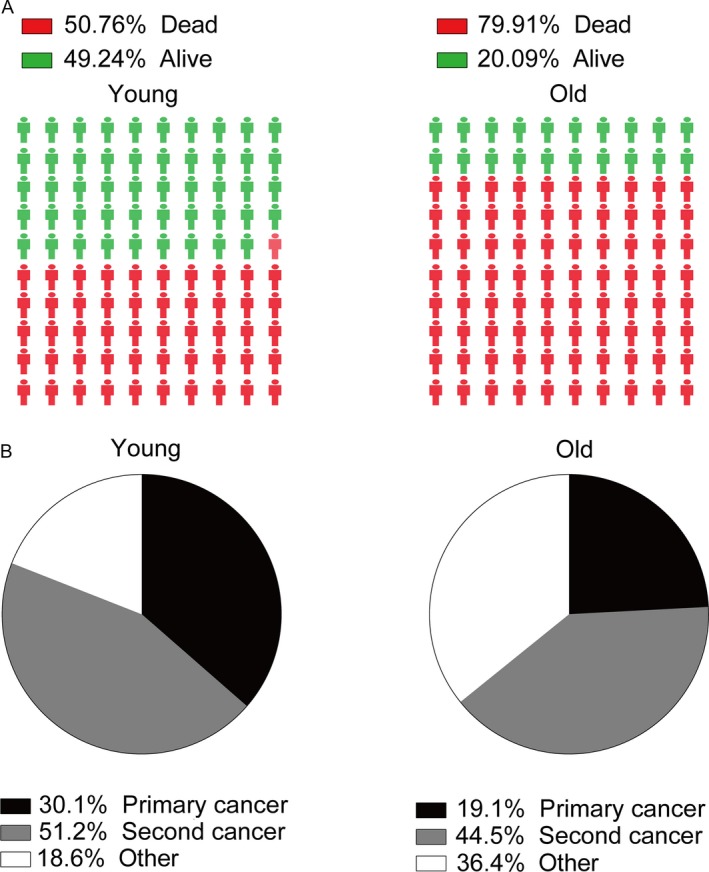
Survival outcomes and cause of death among young and old patients. (A) survival outcomes of young and old patients; (B) cause of death (primary cancer, second cancer, and other causes) among young and old patients *P < 0.05 (compared with general population).

**Figure 6:**
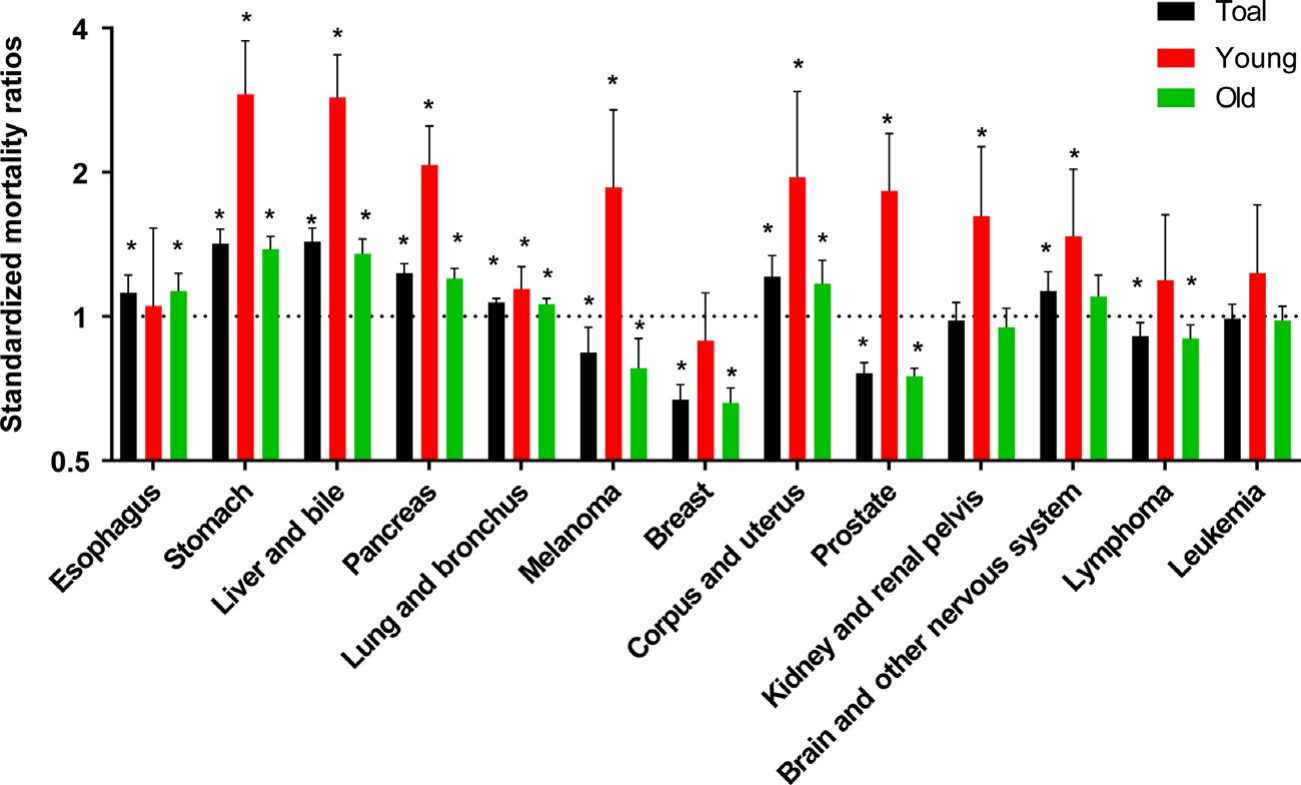
Standardized mortality ratios for secondary cancer types among young and old cancer survivors. *P < 0.05 (compared with general population).

As Figure 5A illustrated that 50.76% of young patients died and more than 79% of old patients died during follow‐up period. As Figure 5B shown, secondary cancers were responsible for 51.2% of all deaths in young survivors, while 44.5% deaths were caused by subsequent cancers in old survivors. Furthermore, we selected several cancer types as common cause of deaths due to SPCs. Compared with general US population, young CRC survivors were at excessive risk of death caused by secondary stomach, liver and bile, pancreas, lung and bronchus, melanoma, corpus and uterus, prostate, kidney and renal pelvis, brain, and other nervous system (Fig. 6). However, a slightly increased risk of death due to secondary esophagus, stomach, liver and bile, pancreas, lung and bronchus, corpus, and uterus was observed (Fig. 6). There was a decreased risk of death due to secondary prostate, breast, melanoma, lymphoma among old groups (Fig. 6).

## Discussion

In view of notable uptrend of young‐onset CRC, growing numbers of young survivors are at risk of SPCs. Our study supported the conclusions of previous literatures that a prior CRC history increased incidence of secondary malignancies 25, 31. In current study, we observed several notable and interesting findings. First, there was a strong inverse association between age and risk of SPCs. The largest SIR and AER were concentrated in aged 20–40 years. Secondly, compared with the US general population, young survivors had a significantly 44% higher SIR of all sites, while the SIR (2%) for old patients was only slightly increased. Thirdly, we provided further detail on the site‐distribution patterns of additional cancers occurring in young survivors. The small intestine, stomach, corpus and uterus, colon, rectum, and bile ducts were the most six common sites for second primary malignancies. Fourthly, significant increased risk of death due to second cancers was observed in young survivors. Compared with general population, young survivors with subsequent stomach, liver and bile, pancreases, and corpus and uterus had higher mortality. Our analysis extends current understanding of the risk of SPCs that young cancer survivors faced.

Previous studies also addressed some aspects of this issue. For example, Liang et al. 27 demonstrated that compared with the general population, young patients in Taiwan (aged <50 years) had higher risk of SPCs (SIR = 2.52, 95%CI, 2.28–2.78), although the SIR for all patients was nonsignificant. Besides, they observed that the most common sites for SPCs in young patients included the small intestine, the large intestine, the female genital organs, and the lungs. In our cohort, we did not observe the significant risk for lung and bronchus in young groups. It might be due to different population. Although the clear reasons for increasing risk of SPCs among young population are not well understood, they may be attributed in part to the genetic and environmental factors, including additional therapy‐related factors, such as radiation and chemotherapy agents 10. Previous studies indicated that patients who received radiation seemed especially prone to develop second malignancies 32. However, in current study, radiotherapy did not significantly increase the risk of all sites SPCs in both young and old patients, except for urinary bladder. As we known, hereditary predisposition and family history may be involved in development of CRC, particularly pronounced for young‐onset CRC, such as Lynch syndrome (also termed hereditary nonpolyposis colorectal cancer, HNPCC). Lynch syndrome is an inherited disorder that is due to defects in DNA mismatch repair genes (such as MLH1, MSH2, MSH6, PMS2, or EPCAM) 33. Those patients were predisposed to develop multiple malignancies in the colorectal, small intestine, stomach, hepatobiliary tract, as well as extradigestive sites including endometrial, ovary, upper urinary tract, brain, and skin 10, 34, 35.

The results presented here should be interpreted cautiously when considering intrinsic limitations of our study. First, due to inherent limitation of SEER database, we were unable to control several cancer risk factors and treatment‐related factors such as chemotherapy, which may introduce some bias. Secondly, we included data from nine registries in SEER because only nine registries were included at first since 1973. Thirdly, we were unable to exclude the possibility of synchronous primary cancers or metastases. Nonetheless, our sensitivity analysis indicated that the overall results were not substantial altered when we excluded patients diagnosed within 1 year of prior CRC (data not shown).

Despite these limitations, our analysis indicated that young CRC survivors had significantly excess risk of SPCs in relative to the general population. Additional studies from other centers need to verify this trend. A comprehensive understanding of the risk faced by young survivors with a history of colorectal cancer would help to determine appropriate prevention strategies. If a true relationship between high risk of SPCs and young age of survivors does exist, young age of onset CRC might imply the need of regular surveillance and monitoring.

## Conflict of Interest

No potential conflict of interests to declare.

## Supporting information


**Table S1**. Cancer site‐specific standardized incidence ratios and absolute excess risk of a Second Primary Cancers (SPCs) for young and old survivor with prior diagnosis of colorectal cancer.
**Table S2**. Cancer site‐specific Standardized Incidence Ratios (SIRs) for second primary cancer by gender.
**Table S3**. Cancer site‐specific Standardized Incidence Ratios (SIRs) for second primary cancer by race.
**Table S4**. Cancer site‐specific Standardized Incidence Ratios (SIRs) for second primary cancer by year.
**Table S5**. Cancer site‐specific Standardized Incidence Ratios (SIRs) for second primary cancer by SEER stage.
**Table S6**. Cancer site‐specific Standardized Incidence Ratios (SIRs) for second primary cancer by colorectal cancer subsite.
**Table S7**. Cancer site‐specific Standardized Incidence Ratios (SIRs) for second primary cancer by latency.
**Table S8**. Cancer site‐specific Standardized Incidence Ratios (SIRs) for second primary cancer by radiation.
