# Supplementary material for: Excessive risk of second primary cancers in young‐onset colorectal cancer survivors
Source: Cancer Med. 2018 Mar 13;7(4):1201–10. doi: 10.1002/cam4.1437 (PMC5911632; doi:10.1002/cam4.1437)
Supplement: Supplementary file 1 — Table S1. Cancer site‐specific standardized incidence ratios and absolute excess risk of a Second Primary Cancers (SPCs) for young and old survivor with prior diagnosis of colorectal cancer. Table S2. Cancer site‐specific Standardized Incidence Ratios (SIRs) for second primary cancer by gender. Table S3. Cancer site‐specific Standardized Incidence Ratios (SIRs) for second primary cancer by race. Table S4. Cancer site‐specific Standardized Incidence Ratios (SIRs) for second primary cancer by year. Table S5. Cancer site‐specific Standardized Incidence Ratios (SIRs) for second primary cancer by SEER stage. Table S6. Cancer site‐specific Standardized Incidence Ratios (SIRs) for second primary cancer by colorectal cancer subsite. Table S7. Cancer site‐specific Standardized Incidence Ratios (SIRs) for second primary cancer by latency. Table S8. Cancer site‐specific Standardized Incidence Ratios (SIRs) for second primary cancer by radiation. [file CAM4-7-1201-s001.docx]

**Supplementary Materials**

**Table S1. Cancer Site-specific Standardized Incidence Ratios and Absolute Excess Risk of a Second Primary Cancers (SPCs) for Young and Old Survivor with Prior Diagnosis of Colorectal Cancer**

| **Cancer site** | **Total patients** | | | **Young patients aged≤50 years** | | |  | **Old patients aged>50 years** | | |
| --- | --- | --- | --- | --- | --- | --- | --- | --- | --- | --- |
|  | **Obs** | **SIR (95%CI)** | **AER** | **Obs** | **SIR (95%CI)** | **AER** |  | **Obs** | **SIR (95%CI)** | **AER** |
| **All sites** | 50,679 | **1.04*(1.03-1.05)** | **8.50** | 3,909 | **1.44*(1.39-1.48)** | **34.23** |  | 46,770 | **1.02*(1.01-1.03)** | **4.29** |
| **All solid Tumours** | 45,897 | **1.07*(1.06-1.08)** | **12.31** | 3,600 | **1.47*(1.42-1.52)** | **33.07** |  | 42,297 | **1.05*(1.04-1.06)** | **8.92** |
| **Haematological diseases** | 3,797 | **0.91*(0.88-0.94)** | **-1.49** | 219 | 1.01 (0.88-1.15) | 0.06 |  | 3,578 | **0.91*(0.88-0.94)** | **-1.75** |
| **Oral cavity and pharynx** | 999 | 0.95 (0.90-1.02) | -0.19 | 56 | **0.72*(0.54-0.93)** | **-0.64** |  | 943 | 0.97 (0.91-1.04) | -0.12 |
| **Esophagus** | 576 | 1.08(1.00-1.17) | 0.18 | 22 | 0.75 (0.47-1.14) | -0.21 |  | 554 | **1.10*(1.01-1.20)** | **0.24** |
| **Stomach** | 1,225 | **1.16*(1.09-1.22)** | **0.67** | 93 | **2.31*(1.86-2.83)** | **1.51** |  | 1,132 | **1.11*(1.05-1.18)** | **0.54** |
| **Small intestine** | 585 | **3.13*(2.89-3.40)** | **1.61** | 104 | **8.49*(6.93-10.28)** | **2.63** |  | 481 | **2.76*(2.52-3.02)** | **1.44** |
| **Colon,** | 7,523 | **1.54*(1.51-1.57)** | **10.65** | 654 | **3.82*(3.53-4.13)** | **13.86** |  | 6,869 | **1.46*(1.42-1.49)** | **10.13** |
| **Rectum** | 2,200 | **1.38*(1.33-1.44)** | **2.47** | 291 | **3.60*(3.20-4.04)** | **6.03** |  | 1,909 | **1.27*(1.21-1.32)** | **1.88** |
| **Liver** | 371 | **0.76*(0.69-0.85)** | **-0.46** | 35 | 0.91(0.63-1.26) | -0.10 |  | 336 | **0.75*(0.67-0.84)** | **-0.52** |
| **Gallbladder** | 106 | **0.65*(0.54-0.79)** | **-0.23** | 5 | 0.93(0.30-2.16) | -0.01 |  | 101 | **0.65*(0.53-0.78)** | **-0.26** |
| **Bile ducts** | 395 | **1.30*(1.18-1.44)** | **0.37** | 46 | **3.77*(2.76-5.03)** | **0.97** |  | 349 | **1.20*(1.07-1.33)** | **0.27** |
| **Pancreas** | 1,478 | 0.99(0.94-1.04) | -0.07 | 102 | **1.62*(1.32-1.97)** | **1.13** |  | 1,376 | 0.96 (0.91-1.01) | -0.27 |
| **Lung and bronchus** | 7,400 | 1.00(0.98-1.03) | 0.08 | 387 | 1.08 (0.97-1.19) | 0.78 |  | 7,013 | 1.00(0.98-1.02) | -0.04 |
| **Bone and joint** | 40 | 1.04(0.74-1.41) | 0.01 | 6 | 1.78 (0.65-3.88) | 0.08 |  | 34 | 0.97(0.67-1.35) | -0.01 |
| **Soft Tissue including Heart** | 210 | 0.94(0.82-1.08) | -0.05 | 24 | **1.59*(1.02-2.37)** | **0.26** |  | 186 | 0.89 (0.77-1.03) | -0.10 |
| **Melanoma** | 1,254 | **0.89*(0.85-0.95)** | **-0.60** | 108 | 0.91 (0.75-1.10) | -0.30 |  | 1,146 | **0.89*(0.84-0.94)** | **-0.65** |
| **Breast** | 4,949 | 0.99(0.96-1.02) | -0.20 | 429 | 0.97 (0.88-1.07) | -0.40 |  | 4,520 | 0.99 (0.97-1.02) | -0.16 |
| **Cervix Uteri** | 166 | 0.96(0.82-1.12) | -0.03 | 24 | 1.06 (0.68-1.57) | 0.04 |  | 142 | 0.95 (0.80-1.12) | -0.04 |

**Table S1** (Continued)

| **Corpus and U****terus** | 1,273 | **1.22*(1.15-1.29)** | **0.93** | 224 | **2.45*(2.14-2.79)** | **3.81** |  | 1,049 | **1.10*(1.04-1.17)** | **0.46** |
| --- | --- | --- | --- | --- | --- | --- | --- | --- | --- | --- |
| **Ovary** | 525 | **0.88*(0.81-0.96)** | **-0.28** | 66 | **1.51*(1.17-1.93)** | **0.64** |  | 459 | **0.83*(0.76-0.91)** | **-0.43** |
| **Prostate** | 8,101 | **0.91*(0.89-0.93)** | **-3.26** | 404 | 0.91 (0.83-1.01) | -1.10 |  | 7,697 | **0.91*(0.89-0.93)** | **-3.61** |
| **Urinary bladder** | 2,947 | 1.00 (0.97-1.04) | 0.04 | 144 | **1.32*(1.12-1.56)** | **1.01** |  | 2,803 | 0.99 (0.95-1.03) | -0.12 |
| **Kidney** | 1,139 | **1.07*(1.01-1.13)** | **0.29** | 110 | **1.37*(1.13-1.65)** | **0.85** |  | 1,029 | 1.04 (0.98-1.11) | 0.20 |
| **Eye and orbit** | 68 | 1.01(0.79-1.29) | 0.00 | 8 | 1.76 (0.76-3.46) | 0.10 |  | 60 | 0.96(0.73-1.24) | -0.01 |
| **Brain** | 360 | **0.85*(0.77-0.95)** | **-0.25** | 34 | 1.06 (0.73-1.48) | 0.05 |  | 326 | **0.84*(0.75-0.93)** | **-0.30** |
| **Thyroid** | 410 | **1.30*(1.18-1.43)** | **0.38** | 81 | **1.48*(1.18-1.84)** | **0.76** |  | 329 | **1.26*(1.13-1.40)** | **0.32** |
| **Lymphoma** | 1,880 | **0.94*(0.90-0.98)** | **-0.49** | 126 | 1.06 (0.89-1.27) | 0.21 |  | 1,754 | **0.93*(0.89-0.98)** | **-0.61** |
| **Myeloma** | 611 | **0.86*(0.79-0.93)** | **-0.40** | 36 | 1.04 (0.73-1.45) | 0.04 |  | 575 | **0.85*(0.78-0.92)** | **-0.48** |
| **Leukaemia** | 1,306 | **0.90*(0.85-0.95)** | **-0.60** | 57 | 0.89 (0.68-1.16) | -0.20 |  | 1,249 | **0.90*(0.85-0.95)** | **-0.66** |
| **Mesothelioma** | 116 | **0.80 (0.66-0.97)** | **-0.11** | 5 | 0.99 (0.32-2.32) | 0.00 |  | 111 | **0.80*(0.66-0.96)** | **-0.13** |

Abbreviation: Obs, Observed events; SIR, Standard incidence ratio; CI, Confidence interval; AER, absolute excess risk. Red colour indicates high risk, blue colour indicates low risk, *P<0.05.

**Table S2.** **Cancer Site-specific Standardized Incidence Ratios (SIRs) for Second Primary Cancer by Gender**

| **Cancer site** | **Young patients aged≤50 years** | |  | **Old patients aged>50 years** | |
| --- | --- | --- | --- | --- | --- |
|  | **Male** | **Female** |  | **Male** | **Female** |
| **All sites** | **1.44*** | **1.44*** |  | **0.99*** | **1.07*** |
| **All solid Tumours** | **1.47*** | **1.48*** |  | 1.01 | **1.11*** |
| **Oral cavity and pharynx** | 0.78 | **0.53*** |  | 1.00 | 0.90 |
| **Esophagus** | 0.64 | 1.20 |  | 1.10 | 1.11 |
| **Stomach** | **2.13*** | **2.69*** |  | **1.11*** | **1.12*** |

**Table S2** (Continued)

| **Small intestine** | **10.54*** | **5.73*** |  | **2.57*** | **3.02*** |
| --- | --- | --- | --- | --- | --- |
| **Colon** | **4.12*** | **3.47*** |  | **1.38*** | **1.54*** |
| **Rectum** | **3.53*** | **3.71*** |  | **1.20*** | **1.37*** |
| **Liver** | 0.92 | 0.87 |  | **0.75*** | **0.76*** |
| **Gallbladder** | 0.00 | 1.39 |  | **0.56*** | **0.69*** |
| **Bile Ducts** | **4.10*** | **3.32*** |  | **1.35*** | 1.01 |
| **Pancreas** | **1.66*** | **1.58*** |  | 0.96 | 0.96 |
| **Lung and bronchus** | 1.07 | 1.09 |  | **0.96*** | **1.08*** |
| **Bone and joint** | 1.56 | 2.09 |  | 0.70 | 1.31 |
| **Soft Tissue including Heart** | 1.16 | **2.18*** |  | 0.94 | 0.82 |
| **Melanoma** | 0.87 | 0.98 |  | **0.89*** | 0.91 |
| **Breast** | 1.55 | 0.96 |  | 1.03 | 0.99 |
| **Cervix Uteri ^a^** | - | 1.06 |  | - | 0.95 |
| **Corpus and Uterus ^a^** | - | **2.45*** |  | - | **1.10*** |
| **Ovary ^a^** | - | **1.51*** |  | - | **0.83*** |
| **Prostate ^b^** | 0.91 | - |  | **0.91*** | - |
| **Urinary bladder** | **1.31*** | 1.37 |  | 0.99 | 1.00 |
| **Kidney** | **1.41*** | 1.30 |  | 1.05 | 1.02 |
| **Eye and orbit** | 2.23 | 1.07 |  | 0.87 | 1.10 |
| **Brain** | 1.29 | 0.71 |  | **0.75*** | 0.96 |
| **Thyroid** | **1.61*** | **1.43*** |  | 1.20 | **1.30*** |
| **Lymphoma** | 1.16 | 0.89 |  | **0.89*** | 0.98 |
| **Myeloma** | 1.15 | 0.92 |  | **0.83*** | **0.88*** |
| **Leukaemia** | 0.93 | 0.82 |  | **0.90*** | **0.89*** |
| **Mesothelioma** | 1.02 | 0.91 |  | **0.79*** | 0.83 |

*P<0.05. a, Cervix Uteri, Corpus and Uterus, Ovary only for female; b, Prostate only for male. Red colour indicates high risk, blue colour indicates low risk.

**Table S3. Cancer Site-specific Standardized Incidence Ratios (SIRs) for Second Primary Cancer by Race**

| **Cancer site** | **Young patients aged≤50 years** | | | |  | **Old patients aged>50 years** | | | |
| --- | --- | --- | --- | --- | --- | --- | --- | --- | --- |
|  | **White** | **Black** | **AI/AN** | **AP** |  | **White** | **Black** | **AI/AN** | **AP** |
| **All sites** | **1.37*** | **1.56*** | **4.08*** | **2.14*** |  | 1.01 | **1.12*** | **1.46*** | **1.17*** |
| **All solid Tumours** | **1.40*** | **1.60*** | **4.24*** | **2.18*** |  | **1.03*** | **1.14*** | **1.48*** | **1.20*** |
| **Oral cavity and pharynx** | **0.71*** | 0.54 | 0.00 | 1.20 |  | 0.95 | 0.93 | 2.00 | **1.56*** |
| **Esophagus** | 0.70 | 1.09 | 0.00 | 0.70 |  | **1.13*** | 0.88 | 1.06 | 1.08 |
| **Stomach** | **2.26*** | 1.48 | **10.80*** | **3.20*** |  | **1.07*** | 1.06 | 0.87 | **1.42*** |
| **Small intestine** | **8.88*** | **6.03*** | 0.00 | **11.95*** |  | **2.68*** | **3.35*** | 3.33 | **3.06*** |
| **Colon** | **3.65*** | **3.58*** | **14.65*** | **5.84*** |  | **1.47*** | **1.47*** | **2.17*** | **1.29*** |
| **Rectum** | **3.28*** | **5.16*** | 4.44 | **4.40*** |  | **1.20*** | **2.23*** | **1.07** | **1.39*** |
| **Liver** | 1.02 | 0.61 | 0.00 | 0.88 |  | **0.71*** | **0.69*** | 0.95 | 0.96 |
| **Gallbladder** | 0.75 | 1.36 | 0.00 | 1.75 |  | **0.62*** | 1.08 | 0.00 | 0.59 |
| **Bile Ducts** | **3.69*** | **5.58*** | 0.00 | **3.37*** |  | **1.20*** | 1.30 | 1.78 | 1.14 |
| **Pancreas** | **1.60*** | 1.74 | 0.00 | 1.88 |  | **0.94*** | 0.97 | 0.60 | **1.27*** |
| **Lung and bronchus** | 1.03 | 1.28 | **3.15*** | 1.26 |  | 0.99 | 1.02 | 1.54 | **1.12*** |
| **Bone and joint** | 1.75 | 0.00 | 0.00 | 5.48 |  | 0.86 | 0.50 | 0.00 | **3.91*** |
| **Soft Tissue including Heart** | **1.56*** | 0.59 | 15.12 | 2.96 |  | 0.89 | 0.98 | 0.00 | 1.03 |
| **Melanoma** | 0.90 | 1.83 | 0.00 | 3.45 |  | **0.89*** | **2.38*** | 2.55 | 0.92 |
| **Breast** | 0.93 | 1.14 | **2.87*** | 1.12 |  | 0.99 | 1.04 | 1.22 | 1.01 |
| **Cervix Uteri** | 1.18 | 0.49 | 6.47 | 0.94 |  | 0.94 | 0.89 | 0.00 | 1.22 |
| **Corpus and Uterus** | **2.30*** | **2.25*** | **8.49*** | **4.23*** |  | **1.09*** | 1.02 | 3.08 | **1.51*** |
| **Ovary** | **1.37*** | 2.19 | **9.92*** | 2.10 |  | **0.83*** | **0.64*** | 1.14 | 1.24 |
| **Prostate** | **0.86*** | 1.14 | 1.30 | 1.26 |  | **0.88*** | **1.07*** | 1.36 | **1.12*** |
| **Urinary bladder** | **1.31*** | 1.38 | 0.00 | 1.82 |  | 0.98 | 1.17 | **2.36*** | 1.14 |
| **Kidney** | **1.42*** | 0.99 | 7.36 | 1.33 |  | 1.01 | **1.30*** | 1.50 | 1.23 |
| **Eye and orbit** | 1.83 | 0.00 | 0.00 | 0.00 |  | 0.96 | 1.77 | 0.00 | 1.02 |
| **Brain** | 0.91 | 2.11 | 0.00 | 3.09 |  | **0.81*** | 1.06 | 1.86 | 1.18 |
| **Thyroid** | **1.47*** | **2.20*** | 0.00 | 1.24 |  | **1.21*** | 1.22 | 1.88 | **1.74*** |
| **Lymphoma** | 1.08 | 0.66 | 0.00 | 1.54 |  | **0.92*** | 0.99 | 1.32 | 1.09 |
| **Myeloma** | 0.95 | 1.26 | 0.00 | 1.60 |  | **0.82*** | 1.03 | 3.34 | 0.89 |

**Table S3** (Continued)

| **Leukaemia** | 0.89 | 1.15 | 0.00 | 0.66 |  | **0.89*** | 0.99 | 1.11 | 1.09 |
| --- | --- | --- | --- | --- | --- | --- | --- | --- | --- |
| **Mesothelioma** | 0.88 | 0.00 | 0.00 | 5.19 |  | **0.79*** | 0.96 | 5.91 | 0.89 |

Abbreviation: AI/AN, American Indian and Alaska Native; AP, Asian or Pacific Islander. *P<0.05. Red colour indicates high risk, blue colour indicates low risk.

**Table S4. Cancer Site-specific Standardized Incidence Ratios (SIRs) for Second Primary Cancer by Year**

| **Cancer site** | **Young patients aged≤50 years** | | | |  | **Old patients aged>50 years** | | | |
| --- | --- | --- | --- | --- | --- | --- | --- | --- | --- |
|  | **1973-1983** | **1984-1993** | **1994-2003** | **2004-2013** |  | **1973-1983** | **1984-1993** | **1994-2003** | **2004-2013** |
| **All sites** | **1.32*** | **1.33*** | **1.51*** | **2.39*** |  | 1.01 | 0.98 | **1.02*** | **1.17*** |
| **All solid Tumours** | **1.34*** | **1.35*** | **1.55*** | **2.46*** |  | **1.04*** | 1.01 | **1.05*** | **1.21*** |
| **Oral cavity and pharynx** | **0.60*** | 0.72 | 0.63 | 1.43 |  | 0.99 | 0.94 | 0.95 | 1.09 |
| **Esophagus** | 0.82 | 0.97 | 0.50 | 0.00 |  | 0.98 | 1.06 | 1.11 | **1.51*** |
| **Stomach** | **2.61*** | **1.85*** | **1.93*** | **3.46*** |  | **1.12*** | 1.06 | **1.26*** | 0.87 |
| **Small intestine** | **7.36*** | **8.08*** | **9.15*** | **12.53*** |  | **2.55*** | **2.28*** | **2.81*** | **4.20*** |
| **Colon** | **3.19*** | **3.62*** | **4.49*** | **7.69*** |  | **1.50*** | **1.32*** | **1.39*** | **2.16*** |
| **Rectum** | **2.29*** | **2.87*** | **3.94*** | **10.83*** |  | 1.05 | 0.94 | **1.33*** | **3.61*** |
| **Liver** | 0.93 | 0.97 | 0.72 | 1.23 |  | **0.64*** | **0.72*** | **0.78*** | 0.89 |
| **Gallbladder** | 1.52 | 0.00 | 1.10 | 0.00 |  | **0.64*** | **0.52*** | 0.79 | 0.76 |
| **Bile Ducts** | **2.68*** | **4.83*** | **3.70*** | **6.20*** |  | 1.16 | **1.23*** | 1.12 | **1.38*** |
| **Pancreas** | **1.62*** | 1.50 | **1.82*** | 1.68 |  | 0.88* | 0.93 | 1.00 | **1.17*** |
| **Lung and bronchus** | 1.00 | 1.02 | **1.32*** | 1.55 |  | **0.91*** | 0.96 | **1.06*** | **1.18*** |
| **Bone and joint** | 1.87 | 1.01 | 2.21 | 2.49 |  | 1.32 | **0.43*** | 0.62 | **2.58*** |

Table S4 (Continued)

| **Soft Tissue including Heart** | 1.14 | 1.78 | 1.33 | **3.24*** |  | **0.65*** | 0.94 | 1.01 | 0.96 |
| --- | --- | --- | --- | --- | --- | --- | --- | --- | --- |
| **Melanoma** | 0.90 | 0.87 | 0.75 | 1.45 |  | **0.85*** | 0.96 | 0.93 | **0.70*** |
| **Breast** | 0.91 | 0.90 | 1.08 | 1.16 |  | 1.01 | 1.04 | **0.93*** | 0.94 |
| **Cervix Uteri** | 0.72 | 1.21 | 1.32 | 1.22 |  | 0.82 | 0.98 | 1.18 | 0.78 |
| **Corpus and Uterus** | **1.98*** | **2.66*** | **2.76*** | **3.01*** |  | 1.02 | **1.12*** | **1.17*** | 1.15 |
| **Ovary** | 1.46 | **1.71*** | 0.90 | **2.69*** |  | 0.95 | 0.88 | **0.74*** | **0.54*** |
| **Prostate** | 0.92 | **0.79*** | 1.02 | 1.31 |  | **0.95*** | **0.89*** | **0.90*** | **0.91*** |
| **Urinary bladder** | **1.30*** | 1.27 | **1.50*** | 1.30 |  | 0.97 | 0.99 | 0.99 | 1.04 |
| **Kidney** | 1.14 | 1.42 | **1.50*** | 1.71 |  | 1.01 | 1.07 | 0.97 | **1.20*** |
| **Eye and orbit** | 1.16 | 2.12 | 1.96 | 2.57 |  | 1.43 | **0.57*** | 1.15 | 0.46 |
| **Brain** | 1.07 | 1.02 | 0.95 | 1.41 |  | 0.88 | 0.85 | **0.79*** | 0.79 |
| **Thyroid** | 1.58 | 1.09 | 1.08 | **2.63*** |  | 0.94 | 1.21 | 1.13 | **1.88*** |
| **Lymphoma** | 1.20 | 1.06 | 0.95 | 0.73 |  | 0.93 | **0.92*** | 0.99 | **0.81*** |
| **Myeloma** | 0.97 | 0.92 | 1.29 | 1.34 |  | 0.87 | **0.85*** | **0.83*** | 0.88 |
| **Leukaemia** | 0.66 | 0.82 | 0.79 | **2.91*** |  | **0.89*** | **0.90*** | 0.91 | 0.89 |
| **Mesothelioma** | 0.75 | 0.00 | 2.96 | 6.14 |  | 0.73 | **0.83** | 0.77 | 0.92 |

*P<0.05. Red colour indicates high risk, blue colour indicates low risk.

**Table S5. Cancer Site-specific Standardized Incidence Ratios (SIRs) for Second Primary Cancer by SEER Stage**

| **Cancer site** | **Young patients aged≤50 years** | | |  | **Old patients aged>50 years** | | |
| --- | --- | --- | --- | --- | --- | --- | --- |
|  | **Localized** | **Regional** | **Distant** |  | **Localized** | **Regional** | **Distant** |
| **All sites** | **1.32*** | **1.54*** | **1.97*** |  | **1.02*** | **1.02*** | **0.94*** |
| **All solid Tumours** | **1.34*** | **1.58*** | **2.04*** |  | **1.05*** | **1.06*** | 0.99 |
| **Oral cavity and pharynx** | 0.77 | **0.61*** | 1.27 |  | 0.96 | 1.00 | 0.95 |
| **Esophagus** | 0.64 | 0.95 | 1.05 |  | **1.14*** | 1.03 | 0.82 |
| **Stomach** | **2.17*** | **2.19*** | **5.72*** |  | **1.16*** | 1.07 | 0.88 |

**Table S5** (Continued)

| **Small intestine** | **5.51*** | **11.90*** | **14.83*** |  | **2.18*** | **3.36*** | **5.62*** |
| --- | --- | --- | --- | --- | --- | --- | --- |
| **Colon** | **3.10*** | **4.52*** | **8.20*** |  | **1.38*** | **1.55*** | **1.64*** |
| **Rectum** | **3.11*** | **3.82*** | **7.01*** |  | **1.18*** | **1.29*** | **2.03*** |
| **Liver** | 0.82 | 0.92 | 2.74 |  | **0.78*** | **0.74*** | 0.56 |
| **Gallbladder** | 1.36 | 0.47 | 0.00 |  | **0.69*** | **0.62*** | 0.20 |
| **Bile Ducts** | **3.33*** | **3.74*** | 2.47 |  | 1.14 | **1.28*** | 1.23 |
| **Pancreas** | **1.47*** | **1.85*** | 1.45 |  | 0.94 | 1.01 | 0.87 |
| **Lung and bronchus** | 0.93 | **1.22*** | 1.33 |  | 0.99 | 1.03 | **0.84*** |
| **Bone and joint** | 1.73 | 1.48 | 0.00 |  | 0.93 | 1.03 | 0.00 |
| **Soft Tissue including Heart** | 1.52 | **1.99*** | 0.00 |  | **0.79*** | 1.02 | 1.18 |
| **Melanoma** | 0.95 | 0.93 | 0.57 |  | 0.96 | **0.83*** | 0.73 |
| **Breast** | 1.02 | 0.88 | 0.95 |  | 1.02 | 0.98 | **0.82*** |
| **Cervix Uteri** | 0.78 | 1.42 | 0.00 |  | 0.88 | 0.99 | 1.90 |
| **Corpus and Uterus** | **2.18*** | **2.85*** | **2.68*** |  | 1.05 | **1.17*** | 1.15 |
| **Ovary** | 1.22 | **1.78*** | **3.19*** |  | **0.78*** | 0.88 | 1.00 |
| **Prostate** | 0.92 | 0.92 | 1.02 |  | **0.96*** | **0.85*** | **0.75*** |
| **Urinary bladder** | 1.00 | **1.69*** | 0.90 |  | 0.99 | 0.99 | 0.95 |
| **Kidney** | **1.43*** | 1.19 | 2.23 |  | 1.08 | 1.01 | 0.92 |
| **Eye and orbit** | 2.50 | 1.10 | 0.00 |  | 0.98 | 1.00 | 0.00 |
| **Brain** | 1.31 | 0.70 | 0.73 |  | 0.91 | **0.79*** | **0.22*** |
| **Thyroid** | 1.36 | **1.59*** | 1.63 |  | **1.25*** | **1.27*** | 1.62 |
| **Lymphoma** | 1.08 | 1.10 | 0.84 |  | 0.95 | **0.93*** | **0.66*** |
| **Myeloma** | 0.91 | 1.24 | 1.72 |  | 0.93 | **0.75*** | 0.72 |
| **Leukaemia** | 0.91 | 0.83 | 1.29 |  | 0.93 | **0.87*** | **0.57*** |
| **Mesothelioma** | 1.10 | 1.00 | 0.00 |  | 0.87 | **0.71*** | 0.24 |

*P<0.05. Red colour indicates high risk, blue colour indicates low risk.

**Table S6.** **Cancer Site-specific Standardized Incidence Ratios (SIRs) for Second Primary Cancer by Colorectal Cancer Subsite**

| **Cancer site** | **Young patients aged≤50 years** | | |  | **Old patients aged>50 years** | | |
| --- | --- | --- | --- | --- | --- | --- | --- |
|  | **Proximal** | **Distal colon** | **Rectum** |  | **Proximal** | **Distal colon** | **Rectum** |
| **All sites** | **1.77*** | **1.33*** | **1.25*** |  | **1.07*** | **1.02*** | **0.96*** |
| **All solid Tumours** | **1.83*** | **1.36*** | **1.26*** |  | **1.10*** | **1.05*** | **0.98*** |
| **Oral cavity and pharynx** | 0.79 | 0.77 | **0.54*** |  | 1.06 | 0.95 | 0.91 |
| **Esophagus** | 0.81 | 0.91 | 0.59 |  | 1.04 | **1.22*** | 0.99 |
| **Stomach** | **3.54*** | **1.80*** | **1.64*** |  | **1.17*** | **1.17*** | 0.96 |
| **Small intestine** | **14.63*** | **6.46*** | **4.73*** |  | **3.69*** | **2.32*** | **1.99*** |
| **Colon,** | **4.80*** | **3.97*** | **2.78*** |  | **1.44*** | **1.60*** | **1.33*** |
| **Rectum** | **4.22*** | **3.27*** | **3.20*** |  | **1.28*** | **1.27*** | **1.22*** |
| **Liver** | 1.35 | 0.72 | 0.77 |  | **0.77*** | **0.82*** | **0.65*** |
| **Gallbladder** | 1.96 | 1.01 | 0.00 |  | **0.65*** | **0.63*** | **0.67*** |
| **Bile Ducts** | **4.96*** | **3.72*** | 2.12 |  | **1.25*** | **1.24*** | 1.07 |
| **Pancreas** | **2.26*** | 1.13 | 1.46 |  | **1.14*** | **0.91*** | **0.79*** |
| **Lung and bronchus** | **1.39*** | **0.75*** | 1.13 |  | 1.02 | **0.94*** | **1.05*** |
| **Bone and joint** | 3.01 | 0.00 | 2.51 |  | 0.97 | 0.58 | 1.36 |
| **Soft Tissue including Heart** | 1.58 | 0.99 | **2.27*** |  | 1.01 | 0.83 | 0.84 |
| **Melanoma** | 0.93 | 0.71 | 1.14 |  | **0.88*** | 0.93 | **0.87*** |
| **Breast** | 1.06 | 0.99 | 0.86 |  | **1.05*** | **0.95*** | 0.97 |
| **Cervix Uteri** | 1.48 | 0.99 | 0.67 |  | 0.92 | 1.02 | 0.92 |
| **Corpus and Uterus** | **3.81*** | **1.94*** | **1.76*** |  | **1.16*** | 1.07 | 1.06 |
| **Ovary** | **2.26*** | **1.65*** | 0.70 |  | **0.86*** | **0.84*** | **0.78*** |
| **Prostate** | 1.01 | 1.06 | **0.69*** |  | 1.03 | **0.95*** | **0.74*** |
| **Urinary bladder** | 1.28 | 1.08 | **1.60*** |  | 0.97 | **0.93*** | **1.09*** |
| **Kidney** | **1.66*** | 1.22 | 1.27 |  | 1.06 | 1.10 | 0.96 |
| **Eye and orbit** | 3.04 | 1.92 | 0.63 |  | 1.11 | 0.93 | 0.86 |
| **Brain** | **1.81*** | 0.73 | 0.62 |  | 0.88 | **0.72*** | 0.93 |
| **Thyroid** | 1.28 | 1.37 | **1.83*** |  | **1.43*** | 1.15 | 1.21 |
| **Lymphoma** | 1.16 | 1.13 | 0.97 |  | 0.98 | **0.84*** | 0.98 |

**Table S6** (Continued)

| **Myeloma** | 1.17 | 0.92 | 1.12 |  | **0.85*** | 0.92 | **0.77*** |
| --- | --- | --- | --- | --- | --- | --- | --- |
| **Leukaemia** | 0.92 | 0.90 | 0.91 |  | 0.92 | 0.93 | **0.85*** |
| **Mesothelioma** | 1.39 | 0.56 | 1.16 |  | **0.65*** | 0.81 | 0.89 |

*P<0.05. Red colour indicates high risk, blue colour indicates low risk,

**Table S7. Cancer Site-specific Standardized Incidence Ratios (SIRs) for Second Primary Cancer by Latency**

| **Cancer site** | **Young patients aged≤50 years** | | | |  | **Old patients aged>50 years** | | | |
| --- | --- | --- | --- | --- | --- | --- | --- | --- | --- |
|  | **Latency (months)** | | | |  | **Latency (months)** | | | |
|  | **6-11** | **12-59** | **60-119** | **120+** |  | **6-11** | **12-59** | **60-119** | **120+** |
| **All sites** | **2.52*** | **2.23*** | **1.55*** | **1.21*** |  | 0.98 | **1.09*** | 1.01 | **0.96*** |
| **All solid Tumours** | **2.59*** | **2.32*** | **1.58*** | **1.23*** |  | 1.02 | **1.13*** | **1.03*** | **0.98*** |
| **Oral cavity and pharynx** | 1.49 | 1.06 | 0.84 | **0.53*** |  | 0.89 | 0.99 | 1.02 | 0.94 |
| **Esophagus** | 0.00 | 0.57 | 0.81 | 0.79 |  | 0.99 | **1.18*** | 1.13 | 1.01 |
| **Stomach** | 1.25 | **2.34*** | **2.67*** | **2.25*** |  | 1.04 | 1.02 | 1.11 | **1.22*** |
| **Small intestine** | **36.08*** | **15.74*** | **7.49*** | **6.30*** |  | **3.61*** | **4.03*** | **1.98*** | **2.03*** |
| **Colon** | **9.77*** | **8.03*** | **4.26*** | **2.84*** |  | **1.73*** | **1.78*** | **1.28*** | **1.22*** |
| **Rectum** | **9.14*** | **8.74*** | **3.51*** | **2.12*** |  | **1.47*** | **1.89*** | 0.93 | **0.75*** |
| **Liver** | 2.91 | 1.11 | 0.39 | 0.97 |  | **0.59*** | **0.77*** | **0.69*** | **0.81*** |
| **Gallbladder** | 0.00 | 0.00 | 1.31 | 1.00 |  | 0.44 | **0.63*** | **0.68*** | **0.67*** |
| **Bile Ducts** | 5.96 | 2.42 | **4.69*** | **3.75*** |  | 1.14 | **1.23*** | **1.33*** | 1.08 |
| **Pancreas** | 2.26 | **2.10*** | **2.28*** | **1.41*** |  | **0.77*** | 0.99 | 0.93 | 0.98 |
| **Lung and bronchus** | 0.80 | **1.68*** | **1.41*** | 0.93 |  | **0.76*** | 1.03 | **1.10*** | **0.92*** |
| **Bone and joint** | 0.00 | 2.54 | 1.41 | 1.73 |  | 0.92 | 1.11 | 0.63 | 1.11 |

**Table S7** (Continued)

| **Soft Tissue including Heart** | 0.00 | 1.04 | **3.12*** | 1.36 |  | 0.89 | 0.90 | 1.05 | **0.77*** |
| --- | --- | --- | --- | --- | --- | --- | --- | --- | --- |
| **Melanoma** | 2.05 | 1.30 | 0.79 | **0.77*** |  | 1.12 | **0.90*** | **0.81*** | 0.92 |
| **Breast** | 0.87 | 1.06 | 0.90 | 0.97 |  | **0.86*** | 1.01 | 0.99 | 1.00 |
| **Cervix Uteri** | 1.69 | 1.32 | 1.10 | 0.76 |  | 1.33 | 1.01 | 1.04 | **0.64*** |
| **Corpus and Uterus** | **5.82*** | **3.61*** | **3.18*** | **1.84*** |  | 1.07 | **1.11*** | 1.09 | 1.10 |
| **Ovary** | **5.07*** | **3.50*** | 1.39 | 0.78 |  | 1.05 | 0.92 | **0.72*** | **0.80*** |
| **Prostate** | 1.17 | **1.41*** | 0.95 | **0.86*** |  | **0.86*** | **0.92*** | **0.93*** | **0.89*** |
| **Urinary bladder** | 0.72 | **1.84*** | **1.69*** | **1.20*** |  | 0.96 | 1.01 | 1.01 | 0.97 |
| **Kidney** | **4.17*** | **2.17*** | 1.31 | 1.11 |  | **1.41*** | **1.14*** | 0.90 | 1.01 |
| **Eye and orbit** | 0.00 | 0.00 | 1.16 | 2.51 |  | 1.06 | 0.82 | 1.11 | 0.97 |
| **Brain** | 0.00 | 1.13 | 1.09 | 1.08 |  | **0.59*** | **0.81*** | 0.92 | 0.84 |
| **Thyroid** | 2.68 | **1.86*** | 1.45 | 1.20 |  | **1.83*** | **1.37*** | 1.23 | 1.03 |
| **Lymphoma** | 1.69 | 1.23 | 1.05 | 1.00 |  | **0.73*** | **0.91*** | 1.00 | 0.93 |
| **Myeloma** | 1.89 | 1.03 | 0.95 | 1.05 |  | **0.64*** | **0.85*** | 0.99 | **0.77*** |
| **Leukaemia** | 0.80 | **1.74*** | 0.89 | 0.73 |  | **0.78*** | **0.83*** | 0.97 | 0.92 |
| **Mesothelioma** | 15.23 | 0.00 | 1.50 | 0.78 |  | 0.56 | 0.83 | **0.66*** | 0.91 |

*P<0.05. Red colour indicates high risk, blue colour indicates low risk,

**Table S8. Cancer Site-specific Standardized Incidence Ratios (SIRs) for Second Primary Cancer by Radiation**

| **Cancer site** | **Young patients aged≤50 years** | |  | **Old patients aged>50 years** | |
| --- | --- | --- | --- | --- | --- |
|  | **Radiation** | **No Radiation** |  | **Radiation** | **No Radiation** |
| **All sites** | **1.47*** | **1.43*** |  | 1.00 | **1.02*** |
| **All solid Tumours** | **1.46*** | **1.47*** |  | 1.01 | **1.05*** |

**Table S8** (Continued)

| **Oral cavity and pharynx** | 0.93 | **0.69*** |  | 1.13 | 0.96 |
| --- | --- | --- | --- | --- | --- |
| **Esophagus** | 0.81 | 0.75 |  | 1.10 | **1.10*** |
| **Stomach** | **3.30*** | **2.19*** |  | 1.08 | **1.12*** |
| **Small intestine** | **6.85*** | **8.79*** |  | **2.74*** | **2.76*** |
| **Colon** | **4.29*** | **3.73*** |  | **1.49*** | **1.45*** |
| **Rectum** | **3.63*** | **3.59*** |  | 1.15 | **1.27*** |
| **Liver** | 0.71 | 0.95 |  | **0.61*** | **0.77*** |
| **Gallbladder** | 0.00 | 1.04 |  | 0.46 | **0.65*** |
| **Bile Ducts** | **4.06*** | **3.75*** |  | 1.23 | **1.20*** |
| **Pancreas** | 1.08 | **1.69*** |  | 0.84 | 0.97 |
| **Lung and bronchus** | 1.31 | 1.03 |  | **1.23*** | **0.97*** |
| **Bone and joint** | 2.05 | 1.75 |  | **2.56*** | 0.81 |
| **Soft Tissue including Heart** | 2.43 | 1.47 |  | 1.31 | **0.85*** |
| **Melanoma** | 1.16 | 0.86 |  | 0.88 | **0.90*** |
| **Breast** | 0.80 | 0.99 |  | 0.93 | 1.00 |
| **Cervix Uteri** | 1.08 | 1.06 |  | 0.49 | 0.98 |
| **Corpus and Uterus** | **2.07*** | **2.50*** |  | **1.92*** | 1.04 |
| **Ovary** | 0.40 | **1.66*** |  | 0.91 | **0.83*** |
| **Prostate** | **0.63*** | 0.96 |  | **0.47*** | **0.96*** |
| **Urinary bladder** | **2.51*** | 1.17 |  | **1.43*** | **0.95*** |
| **Kidney** | 1.18 | **1.41*** |  | 0.92 | 1.05 |
| **Eye and orbit** | 0.00 | 2.05 |  | 0.71 | 0.97 |
| **Brain** | 0.69 | 1.12 |  | **0.64*** | **0.86*** |
| **Thyroid** | 1.20 | **1.50*** |  | 1.39 | **1.25*** |
| **Lymphoma** | 1.35 | 1.03 |  | 1.07 | **0.92*** |
| **Myeloma** | 0.73 | 1.09 |  | **0.61*** | **0.88*** |
| **Leukaemia** | 1.50 | 0.81 |  | 0.91 | **0.90*** |
| **Mesothelioma** | 0.00 | 1.13 |  | 0.70 | **0.81*** |

*P<0.05. Red colour indicates high risk, blue colour indicates low risk,
